# Genome sequences of *Bacillus spizenii* SHT-15 isolated from cotton (*Gossypium hirsutum*) rhizosphere in the arid region of Northwest China

**DOI:** 10.1128/mra.00536-25

**Published:** 2026-01-26

**Authors:** Zhichao Meng, XinXiang Niu, Ablimit Nuraliya, Yue Sheng, Hongmei Yang, Ming Chu, Ning Wang, Huifang Bao, Faqiang Zhan, Rong Yang, Kai Lou, Shuang Dou, Zhao Zhang, Yun Chen, Yignwu Shi

**Affiliations:** 1 College of Life and Science and Technology, Xinjiang University, Urumqi, Xinjiang, China; 2 Institute of Microbiology, Xinjiang Academy of Agricultural Sciences 74608, Urumqi, Xinjiang, China; 3 Xinjiang Laboratory of Special Environmental Microbiology, Urumqi, Xinjiang, China; 4 Institute of Agricultural Resources and Environment, Xinjiang Academy of Agricultural Sciences, Urumqi, Xinjiang, China; 5 Key Laboratory of Agricultural Environment in Northwest Oasis of Ministry of Agriculture and Coun-tryside, Urumqi, Xinjiang, China; 6 College of Agronomy, Xinjiang Agricultural University, Urumqi, Xinjiang, China; Wellesley College, Wellesley, Massachusetts, USA

**Keywords:** *Bacillus spizenii*, cotton *Verticillium *wilt, antibacterial ability, coding sequence, genome sequencing

## Abstract

*Bacillus spizenii* strain SHT-15 was isolated from the rhizosphere soil in Shihezi, Xinjiang, China. This study presents the whole-genome sequencing of strain SHT-15, revealing a genome size of 4.082 Mb, which comprises 4,185 predicted protein-coding sequences and 96 RNA genes.

## ANNOUNCEMENT

*Bacillus spizenii*, a gram-positive bacterium, is renowned for secreting antimicrobial peptides and other substances inhibiting plant pathogens (1). It also acts as a biosurfactant, enhancing anti-pathogenic efficacy. The *B. spizenii* is extensively studied in molecular and cell biology due to its large genome and genetic versatility (2, 3).

In Shihezi City, Xinjiang, China, the rhizosphere antagonistic bacterium SHT-15 was isolated from healthy cotton plants to control cotton verticillium wilt (4). Using the dilution plating method, soil samples were diluted to 10^−6^, spread onto TSA medium plates, and incubated at 33°C for 24 h. The antibacterial activity was determined by the plate confrontation method, and dominant antagonistic strains were purified on nutrient agar medium. Pot experiments with a 2×10^8^ CFU/mL bacterial suspension showed that SHT-15 achieved a 89.23% control efficiency against tomato root rot caused by *Fusarium oxysporum* during the seedling stage.

The 16S rRNA gene sequence similarity was calculated using DNAMAN 8.0 software with primers 27F (5-AGAGTTTGATCCTGGCTCAG-3) and 1492R (5- ACGGCTACCTTGTTACGACTT-3), revealing a 99.52% identity with *B. spizenii* (NR_112686.1) (5). Additionally, a BLAST search in the NCBI NT database confirmed that the closest matching sequence was *B. spizenii* (NR_112686.1).

Genomic DNA of *B. spizenii* SHT-15 was extracted using the Ezup Column Bacterial Genomic DNA Extraction Kit. Bioinformatics analysis was performed on data from the Illumina platform. Quality checks and trimming were conducted using FastQC v0.11.7 (6) and Trimmomatic v0.39 (7). The clean short reads were assembled into complete genomes using SOAPdenovo v. 2.04 (8) and polished with Pilon v1.22 (9), and Quast v5.0.2 (10) evaluated the genome assembly quality. CheckM v1.1.6 (11) assessed completeness and contamination. The draft genome was annotated using the Rapid Annotation System Technology (RAST) (12) Pipeline and NCBI PGAP v6.5 (13) and assessed against the Genome Taxonomy Database using GTDB-Tk v1.7.0 (14).

**Fig 1:**
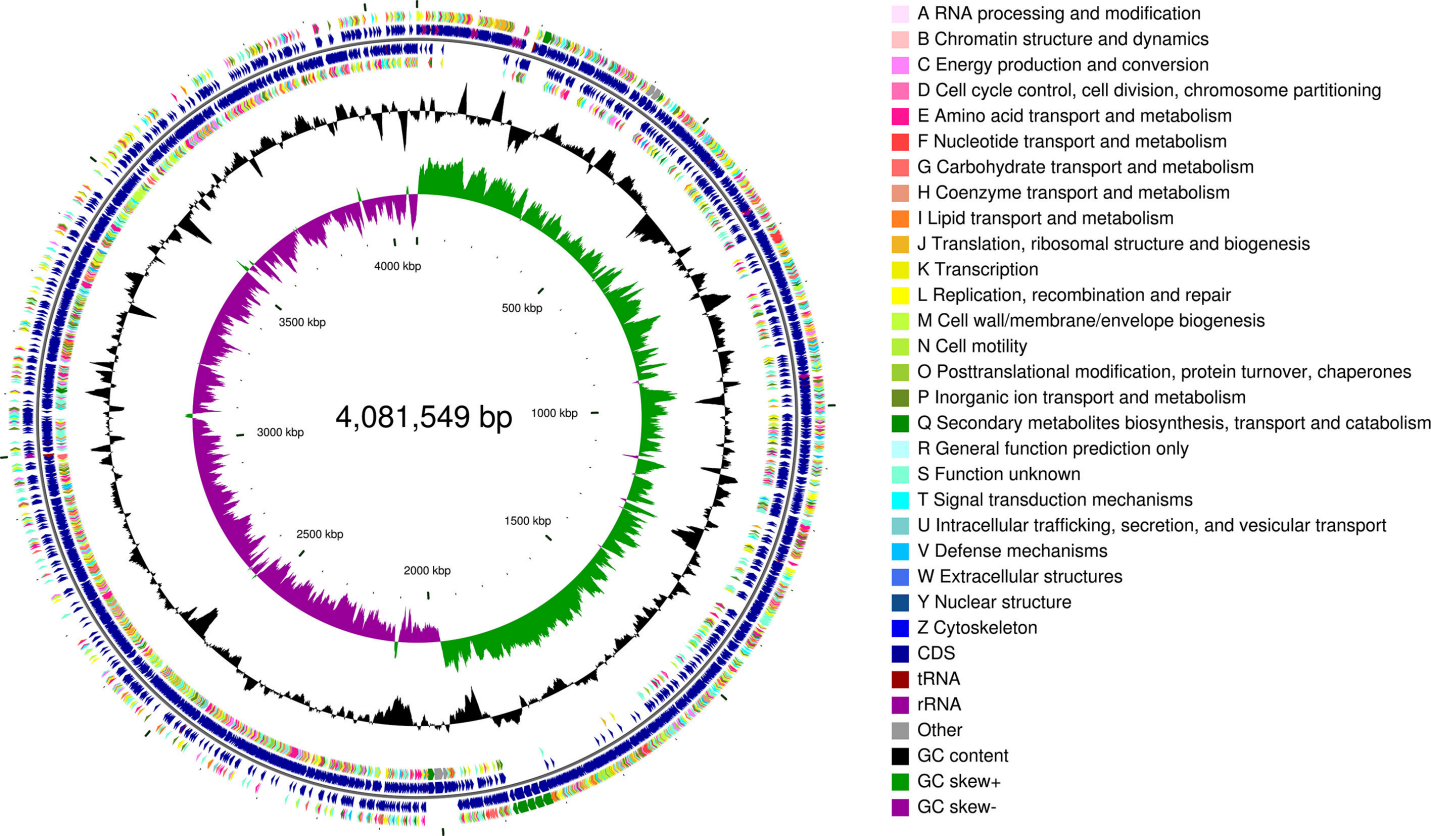
Genome-wide mapping of *B. spizenii* SHT-15. Note: The outermost circle of the circle graph is the identification of genome size; the second and third circles are CDS on the positive and negative chains, and different colors represent the functional classification of different COGs of CDS; the fourth circle is rRNA and tRNA; the fifth circle is GC content. The outward red part indicates that the GC content in this region is higher than the average GC content of the whole genome. The higher the peak value is, the greater the difference between the average GC content is. The inward blue part indicates that the GC content in this region is lower than the average GC content of the whole genome. The higher the peak value is, the greater the difference between the average GC content and the average GC content is. The innermost circle is the GC-Skew value, and the specific algorithm is G-C / G+C, which can assist in judging the leading chain and the lagging chain. In general, the leading chain GC skew > 0, the lagging chain GC skew.

The whole genome of *B. spizenii* SHT-15 consisted of a 4,081,549 bp chromosome with 279,920 reads, 3,920 protein-coding sequences, 30 rRNA genes, 86 tRNA genes, and a G+C content of 44.69%. Among 4,034 CDS, 767 are associated with transporters, 1,132 encode transmembrane proteins, and 421 relate to virulence, potentially explaining its inhibitory effect on cotton verticillium wilt (Fig. 1). Future studies will investigate the inhibition mechanism of *B. spizenii* SHT-15 on cotton verticillium wilt.

## Data Availability

The whole-genome shotgun project for *B. spizenii* SHT-15 has been deposited at DDBJ/ENA/GenBank under the accession CP167793, and the version described in this paper is version CP167793. The raw reads are available under the BioProject accession number PRJNA1149500, and the BioSample accession number is SAMN43249788. The sequence data obtained in this work have been deposited in the NCBI Sequence Read Archive under the accession number SRR33847918. Additionally, the assembled genome sequence is available in GenBank under accession number GCA_041501465.1.
